# Design and Preparation of CNTs/Mg Layered Composites

**DOI:** 10.3390/ma15030864

**Published:** 2022-01-23

**Authors:** Xiao Zhang, Linchi Zou, Junfeng Chen, Pinqiang Dai, Jian Pan

**Affiliations:** 1 College of Materials Science and Engineering, Fujian University of Technology, 3 Xueyuan Road, University Town, Fuzhou City 350118, China; zhangxiao19940629@163.com (X.Z.); pqdai@126.com (P.D.); pj18756863240@163.com (J.P.); 2 Fujian Provincial Key Laboratory of Advanced Materials Processing and Application, 3 Xueyuan Road, University Town, Fuzhou City 350118, China; 3 School of Materials Science and Engineering, Qishan Campus, Fuzhou University, 2 Xueyuan Road, University Town, Fuzhou City 350116, China

**Keywords:** electrophoretic deposition (EPD), spark plasma sintering (SPS), composites, mechanical properties, damping properties

## Abstract

In order to effectively solve the problem of strength and ductility mismatch of magnesium (Mg) matrix composites, carbon nanotubes (CNTs) are added as reinforcement. However, it is difficult to uniformly disperse CNTs in a metal matrix to form composites. In this paper, electrophoretic deposition (EPD) was used to obtain layered units, and then the CNTs/Mg layered units were sintered by spark plasma sintering to synthesize layered CNTs/Mg composites. The deposition morphology of the layered units obtained by EPD and the microstructure, damping properties, and mechanical properties of the composite material were analyzed. The results show that the strength and ductility of the composite sample sintered at 590 °C were improved compared with the layered pure Mg and the composite sample sintered at 600 °C. Compared with pure Mg, the composites rolled by 40% had a much higher strength but no significant decrease in ductility. The damping properties of the CNTs/Mg composites were tested. The damping–test-temperature curve (tanδ~T) rose gradually with increasing temperature in the range of room temperature to 350 °C, and two internal friction peaks appeared. The damping properties of the tested composites at room temperature decreased with increasing frequency. The layered structure of the CNTs/Mg had ultra-high strengthening efficiency and maintained its ductility. The layered units prepared by EPD can uniformly disperse the CNTs in the composites.

## 1. Introduction

Mg is one of the lightest structural metals, and it is distinguished by very good specific strength and specific stiffness, the best damping properties among various metallic materials [1,2]. Combining Mg with other materials into composites can maximize the advantages of various materials and obtain materials with excellent comprehensive performance [3,4,5]. However, only the introduction of conventional reinforcing phases can increase the strength and decrease the ductility simultaneously, which will cause serious and unpredictable failures. Thus, it will be limited in industrial applications [6,7,8].

M.S. Strano et al. [9] found that if the size of the reinforcement is reduced to the nanometer scale and the reinforcement can be dispersed uniformly, then the strength of Mg matrix composites can be improved while maintaining its plasticity. CNTs have good mechanical properties such as high tensile strength, low density, and high elastic modulus. Therefore, CNTs with a variety of performance advantages are considered to be ideal reinforcement materials for the manufacture of nanocomposites [10]. The application of CNTs to enhance the mechanical properties of Mg matrix composites faces two challenges. First, due to the nano effect of the CNTs themselves, the CNTs easily agglomerate and cannot be evenly distributed in the metal matrix. Second, the lack of sufficient interface bonding between the CNTs and the matrix will greatly affect the performance of the final material [11].

Microlaminated materials, ultrafine layered structural materials prepared by the bionic design of shell structures, are composed of submicron calcium carbonate layers and alternately arranged nano-level organic layers, which have the dual functions of strengthening and toughening [12]. The composite prepared by amination technology builds a micro-/nanolaminated structure similar to shell nacre. The strength and elastic modulus of the material are not only greatly improved but also show excellent plasticity [9,12,13]. However, biomimetic laminated Mg matrix composites still have unresolved problems, especially the preparation method and strengthening and toughening mechanism. Because the CNTs/Mg laminated structure has ultra-high strengthening efficiency, this structure is regarded as the main cause of high back stress, but the toughening mechanism has not been studied [14]. 

At present, research on the damping of Mg matrix composites mainly focuses on the material system, thermal processing, and preparation technology [15]. Test parameters, such as strain amplitude, temperature, and vibration frequency, will also have a certain impact on the damping performance of Mg matrix composites [16]. Although many studies have been conducted on the damping behavior of Mg matrix composites, there are still ambiguities regarding temperature-dependent damping capabilities. The study found that the damping of CNTs/Mg composites comes mainly from the following aspects: (1) pure Mg and CNTs have high damping properties, so their intrinsic damping is the source of CNTs/Mg composites; (2) the thermal expansion coefficient mismatch between matrix and reinforcement material, where the high-density dislocation produced by MMC processing has a significant impact on the loss of elastic strain energy, thus increasing its damping capacity [3]; (3) the mobility of the discontinuous microstructure and interface slip at the interface of pure Mg and CNTs results in interface damping [17].

In this study, to effectively solve the problem of mismatch between the strength and ductility of Mg matrix composites and the uniform dispersion of CNTs in the Mg matrix, CNTs/Mg composites were prepared by the biomimetic lamination method. The preparation scheme was as follows: First, the CNTs were pretreated by the acid mixing method and ultrasonic dispersion method. Then, carbon nanoreinforcements were evenly distributed on Mg foil by EPD, and the combination of different layers was realized by SPS. Finally, the tensile and damping properties of the prepared samples were tested, and the morphology, tensile fracture morphology, and metallographic images of the layered structure of CNTs deposited on Mg foil were observed.

## 2. Experimental Method

**Figure 1:**
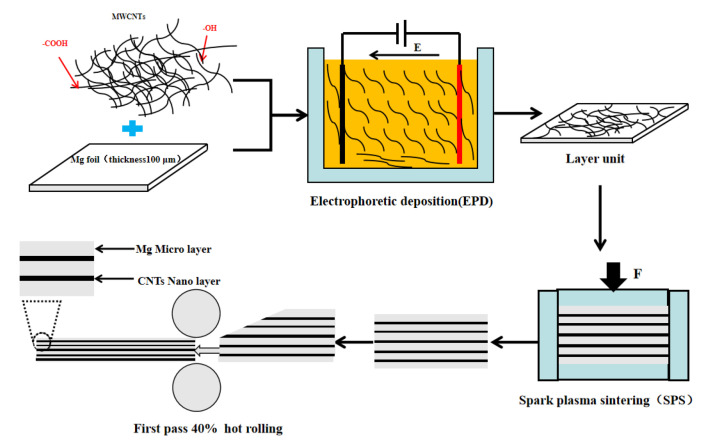
Schematic diagram of the fabrication process of CNTs/Mg laminated composites.

Figure 1 shows the preparation process of CNTs/Mg biomimetic laminated composites including EPD and spark plasma sintering (SPS). First, EPD was used to obtain CNTs/Mg layer units. Then, the layer units were stacked and sintered by spark plasma to synthesize bulk composites. After, the composites were rolled at room temperature.

### 2.1. Preparation Process

#### 2.1.1. CNTs’ Pre-Dispersion Treatment

Multi-walled CNTs (diameter: 10–20 nm, length: 5–15 μm) were supplied by Shenzhen Nanotech Port Co., Ltd., (Shenzhen, China). To enhance the dispersibility of CNTs in isopropanol, oxygen-containing functional groups were introduced on the surface of the CNTs. The CNTs were added to a mixed acid solution of H_2_SO_4_ and H_2_O_2_ (volume ratio of 7:3). After ultrasonic vibration at room temperature for 2 h, the mixed solution was repeatedly diluted and filtered to pH = 7 and then dried at 110 °C.

#### 2.1.2. Preparation of Layered Units by Electrophoretic Deposition

The EPD suspension was composed of 0.4 g/L Al(NO_3_)_3_ and 0.2 g/L functioning CNTs in isopropanol with 6 h ultrasonic dispersion. The cathode of the EPD was Mg foil (100 mm × 100 mm × 100 μm). The anode was a stainless-steel sheet of the same size. The EPD voltage was 300 V with a 6 cm distance between two electrodes. The CNT content on the Mg foil was controlled by the deposition times, which were 0.5, 1, and 1.5 min.

#### 2.1.3. Spark Plasma Sintering

The CNTs/Mg foil obtained by EPD was stacked into approximately 46 layers, and then they were placed in a 30 × 30 mm square hole high-strength graphite mold for SPS. The schematic diagram of sintering is shown in Figure 1. The sintering temperatures of the samples were 590 and 600 °C, respectively.

### 2.2. Materials Characterization

The morphology of CNTs deposited on the surface of Mg foil with the fracture morphology of tensile samples was observed by a field emission scanning electron microscope (FE-SEM, Nova Nano SEM 450, FEI, Hillsborough, OR, USA). Transmission electron microscopy (TEM, Tecnai F20, FEI, Hillsborough, OR, USA) was used to study the CNTs/Mg interface and CNTs in laminated composites. The structure of pickled CNTs was characterized by Raman spectroscopy. The laser wavelength selected in the experiment was 514.5 nm. The phase determination was carried out with X-ray diffractometer (XRD, D8 ADVANCE, Bruker AXS, Karlsruhe, Germany). X-ray photoelectron spectroscopy (XPS, ESCALAB 250, Thermo Fisher Scientific, Waltham, MA, USA) was used to characterize the types of functional groups and the content of various elements on the surface of CNTs after acid washing.

**Figure 2:**
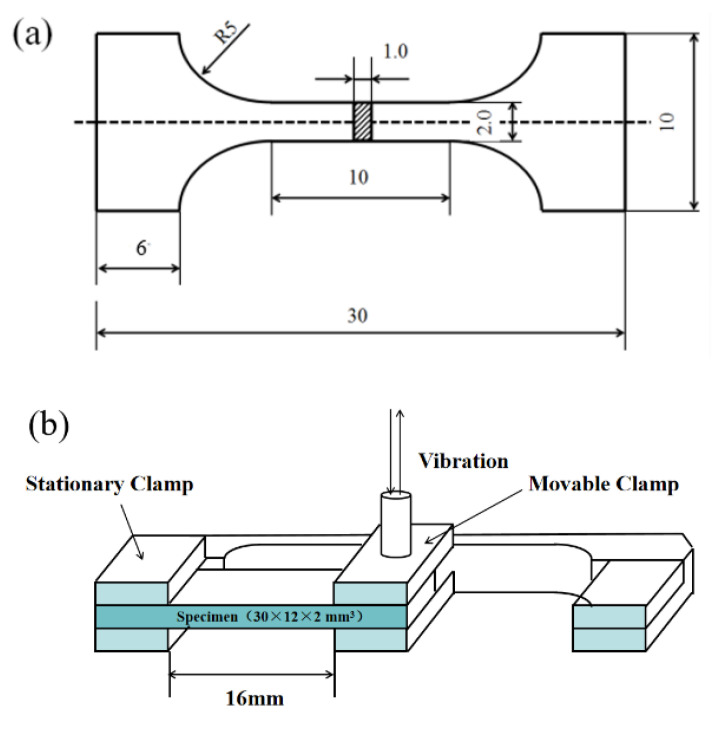
(**a**) The shape and size of tensile specimens; (**b**) schematic diagram of the single cantilever clamp.

The tensile properties were tested by an electronic universal testing machine (Instron 2382, Instron, Boston, MA, USA). The crosshead speed was 0.6 mm/min. Dog-bone-shaped specimens with a gauge length of 10 mm, width of 2 mm, and thickness of 1 mm were used for tensile testing (Figure 2a). Three samples of each type of material were tested to obtain the yield strength (YS), ultimate tensile strength (UTS), and elongation.

The damping behavior of CNTs/Mg was observed by dynamic mechanical analysis (DMA 242E, NETZSCH, Selb, Germany). The damping test adopted a single cantilever mode, and the fixture structure is shown in Figure 2b. Test sample size (L × w × h) were 30 × 12 × 2 mm^3^. Test conditions for this damping test: (1) the test conditions of the damping-frequency spectrum at room temperature were 0.5, 1, 2, 5, and 10, and the strain amplitude was 30 × 10^−6^; (2) the test conditions of the damping-temperature spectrum were a frequency of 1 Hz, test temperature of 25–350 °C, heating rate of 2 °C/min, and a strain amplitude of 30 × 10^−6^.

## 3. Results and Discussion

### 3.1. The Structure of the CNTs Treated with Mixted Acid

**Figure 3:**
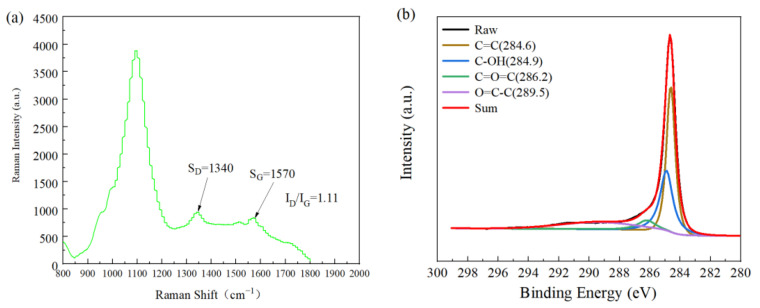
(**a**) Raman spectrum of the pretreated CNTs; (**b**) analysis of the C_1s_ peak position by CNTs XPS after mixed acid treatment.

Figure 3a shows the Raman spectra of CNTs after mixed acid treatment. There were two characteristic peaks at 1340 and 1570 cm^−1^, which are the D peak and G peak, respectively. The D peak was related to irregular carbon atoms with hanging bonds at the edge of the two-dimensional plane that could reflect the disordered arrangement of the graphite layers. The G peak corresponded to the E_2G_ vibration mode of graphite, such as the SP^2^ bond of the graphite layer, which is related to the vibration of the SP^2^ bond carbon atom in two dimensions. The hexagonal lattice could reflect the structural characteristics of the original graphite. Therefore, the ratio of the D peak to the G peak (I_D_/I_G_) reflected the structural integrity of CNTs [6]. For CNTs with the original structure, the intensity ratio (I_D_/I_G_) of the D-band and G-band peaks was approximately 0.99 [18], while the intensity ratio (I_D_/I_G_) of the two bands of pretreated CNTs was approximately 1.11 as shown in Figure 3. The numerical value increased by 12%, indicating that the CNT structure was damaged to a certain extent, and a certain number of functional groups were produced on the surface. The larger the value, the greater the damage to the CNT structure caused by mixed acid treatment and the more functional groups generated on the surface, which is conducive to the adsorption of metals and compounds by CNTs.

In Figure 3b, the band spectrum of XPS C_1s_ shows that after acid treatment, peaks appeared at 284.9, 286.2, and 289.5 eV, corresponding to hydroxyl, carbonyl, and carboxyl functional groups, respectively. The existence of these functional groups, on the one hand, acts as a bridge to improve the wettability of CNTs in the matrix; on the other hand, CNTs in suspension can be effectively dispersed to delay agglomeration.

### 3.2. Morphology of CNTs Electrodeposited on Mg Foil

**Figure 4:**
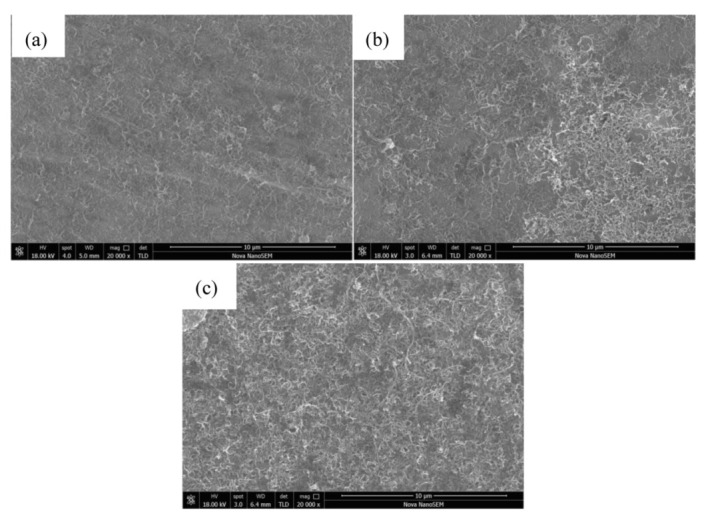
The SEM images for CNTs deposited on Mg foils with different EPD times: (**a**) 0.5; (**b**) 1; (**c**) 1.5 min.

As shown in Figure 4a, through EPD, CNTs were uniformly deposited on the surface of the Mg foil, and agglomeration of CNTs was not observed. Therefore, the application of EPD can achieve a uniform distribution of CNTs on the surface of Mg foil. Oxygen-containing functional groups were introduced into the surface of CNTs treated with mixed acid to uniformly disperse the CNTs in the EPD suspension [7].

In addition, the EPD time was a decisive factor affecting the deposition morphology at 300 V. If the deposition time is too long, due to the excellent conductivity of CNTs, a reverse electric field will form in the electrophoresis fluid after deposition. In this way, the deposition current in the experiment will weaken as time goes on, and the CNTs deposited after disturbance will drive the CNTs deposited first to fall off and the temperature of the electrophoretic fluid will rise, and the thermal movement of ions in the electrophoretic fluid will be intensified. The above two conditions will cause an uneven deposition thickness of CNTs on the surface of the matrix. In addition, excessive deposition of CNTs will lead to agglomeration of nanolayers, as shown in Figure 4b,c, resulting in defects that result in poorer material properties than expected.

The evaluation criteria for the deposition effect of CNTs on the surface of Mg foil with different deposition times (i.e., 0.5, 1, and 1.5 min) under 300 V using SEM were as follows: (1) the uniform distribution of CNTs and large gaps were conducive to the effective bonding of the interlayer Mg foil, but the content of the CNTs was too small and the content of the reinforcement was too small as well, which may have affected the overall properties of the composites; (2) CNT layers were densely stacked, and some CNTs overlapped, but a sufficient gap was still reserved, and the deposition was in good condition; (3) there were undispersed agglomerates deposited on the surface of the Mg foil. These agglomerates were soft and had no binding force. They could not transfer loads in the composite and were the nucleation point of the crack.

The EPD solution that was used to prepare a batch of samples was put into a vacuum drying oven for drying, the mass of the remaining CNTs were weighed, and the mass of the remaining CNTs was subtracted from the mass of the CNTs added to the electrophoretic solution to calculate the mass of the CNTs deposited on the Mg foil. The mass percentage of CNTs in the sample was calculated by dividing the mass of the remaining CNTs by the mass of the composite. Through SEM observation of the CNTs deposited on the Mg foil, it could be seen that the morphology of 1 and 1.5 min did not meet our requirements. Therefore, this paper mainly studied the parameters of 300 V and 0.5 min. Therefore, through the above scheme, it was calculated that the content of the CNTs in the sample was approximately 0.018%.

### 3.3. Mechanical Properties of CNTs/Mg Laminated Composites

**Figure 5:**
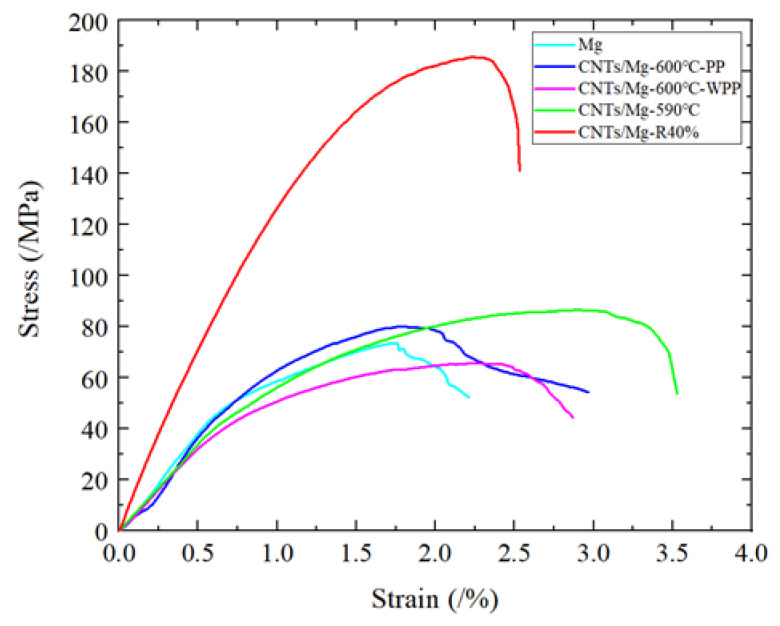
Engineering tensile stress–strain curves of rolled CNTs/Mg composites and CNTs/Mg composites.

Figure 5 shows the stress–strain curves of the pure Mg and CNTs/Mg layer composites. Detailed values of the tensile properties are given in Table 1. Under the condition of sintering at 600 °C and preserving pressure to room temperature, for the layered pure Mg compared with the biomimetic layered CNTs/Mg, the UYS and elongation increased by 8.3% and 15%, respectively. Compared with pure Mg, CNTs/Mg composites further improved the UYS but did not reduce the ductility. However, after the same sintering temperature mentioned above, the UTS of the sample without preservation pressure to room temperature (CNTs/Mg-600 °C-WPP) decreased but the elongation increased compared with the sample preserve pressure to room temperature (CNTs/Mg-600 °C-PP). Compared with the samples sintered at 590 °C, the UTS and elongation increased by 32% and 65%, respectively. The UTS of the composites with 40% rolling deformation of the samples (CNTs/Mg-R40%) sintered at 590 °C even reached 185 MPa, which was far better than the UTS of pure Mg. In summary, the CNTs/Mg layer structure showed good strengthening and toughening effects. The micro/nanostructure balances the conflict between strength and ductility of the Mg matrix composites. Compared with other Mg matrix composites [19,20,21,22,23,24,25,26], the CNTs/Mg layered composites could not only achieve a good strengthening effect but also had an excellent toughening effect.

#### 3.3.1. Enhancement Mechanism of the CNTs/Mg Composite

**Figure 6:**
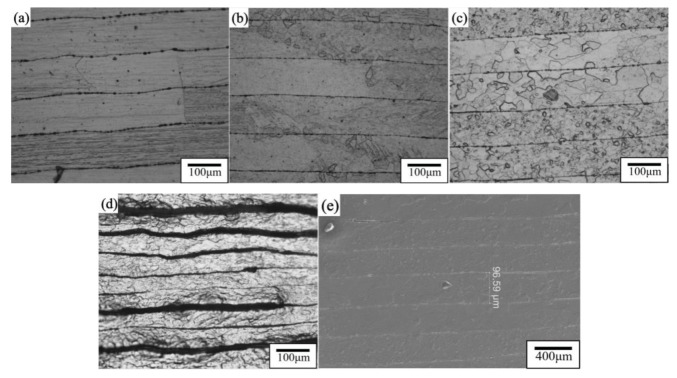
The lateral layered metallographic image and electron microscope image of CNTs/Mg composite material: (**a**) 600 °C sintering; (**b**) 600 °C sintering without preserving pressure after the end of holding time; (**c**) 590 °C sintering; (**d**) 590 °C sintering after the pass 40% hot rolling; (**e**) SEM of the 590 °C sintered sample picture.

The strengthening efficiency of the CNT layer in CNTs/Mg composites greatly exceeds the simple mixing rule. Compared with traditional MMCs, the simultaneous interpreting mechanism usually includes the following parts: grain refinement mechanism (Δσ_Hall Petch_), load transfer from the matrix to the reinforcement (Δσ_LT_), and dislocation increase by thermal expansion coefficient (CTE) mismatch (Δσ_CTE_) generation [14]. As shown in Figure 6, almost all CNTs were distributed at the grain boundaries, so Orowan strengthening could be ignored.

**Figure 7:**
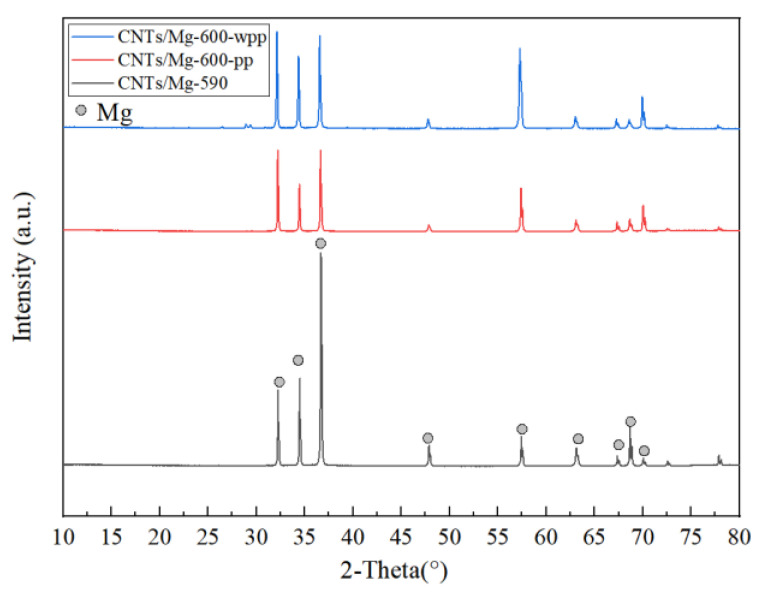
XRD pattern of the materials.

The typical XRD patterns of the CNTs/Mg composites at different temperatures and pressurization methods are shown in Figure 7. The distribution of the composites included peaks corresponding to the Mg phase. The peak of Mg–C was not clearly shown in the detected results, which indicates that there was no reaction between Mg and C.

**Figure 8:**
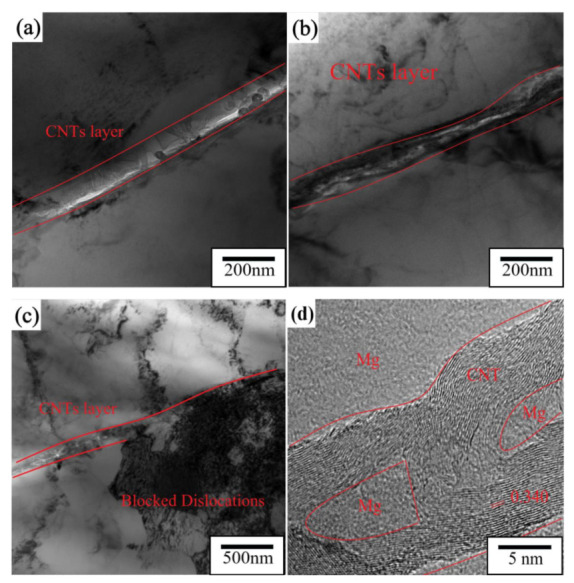
TEM image of CNTs/Mg composite material: (**a**) CNTs were dispersed in a line and some of them were embedded in the matrix; (**b**) CNTs were dispersed in a line; (**c**) dislocations accumulated around the CNTs; (**d**) HRTEM image of the CNTs/Mg interface.

Compared with traditional reinforcement materials, the huge aspect ratio of CNTs makes load transfer more efficiently [14]. Shear lag models have widely been used to predict load transfer mechanisms. The contribution of the load transfer mechanism to the yield strength can be estimated by equation [27]. In the process of tensile strain, the Mg matrix is the main cause of plastic deformation, and the plastic deformation behavior of the Mg matrix is greatly affected by the layered structure simultaneously. When the sintering temperature was 600 °C, the crystal grains in the Mg layer of the sample after 6 min of heat preservation at 600 °C were held to room temperature at a pressure of 30 MPa and were mainly rectangles and bricks. The grain boundaries overlapped with the CNTs/Mg interface, and the short edges of the rectangular grains were usually along the interlayer direction. The high-strength CNT layer effectively blocked the growth of the grains along the normal direction (ND) [28] as shown in Figure 8. Nano-layered CNTs were combined well with the micro-layered Mg matrix as shown in Figure 8d. The metallographic diagram of the sample that was relieved of pressure after 6 min of heat preservation at 600 °C shows that some of the matrix layers had fine grains, but most of the grains of the matrix layer were mainly rectangles and bricks as shown in Figure 6b. At the same sintering temperature, the matrix grains of the sample that were kept pressed to room temperature were larger than those that were not kept pressed to room temperature. When the sintering temperature was reduced to 590 °C, some of the Mg matrix layers had smaller crystal grains, some of the Mg matrix layers had relatively large crystal grains, and the grain size distribution was uneven as shown in Figure 6c. This indicates that the decrease in temperature can effectively prevent the grain size from becoming too large, but the decrease in sintering temperature makes the combination between Mg and CNTs insufficiently dense, which may lead to material scattering due to the poor combination in the subsequent preparation process. After hot rolling at 590 °C for 40% of the first pass (hot rolling temperature: 350 °C), the number of fine grains was more than that of sintered samples, the grain size difference between each matrix layer was not too large, and the spacing between CNT layers was reduced from 100 to 60 μm as shown in Figure 6d. After rolling, not only did the grain size in the Mg matrix decrease but the distribution of CNTs on the nanolayer also became more uniform. 

#### 3.3.2. Toughening Mechanism of the CNTs/Mg Composite

**Figure 9:**
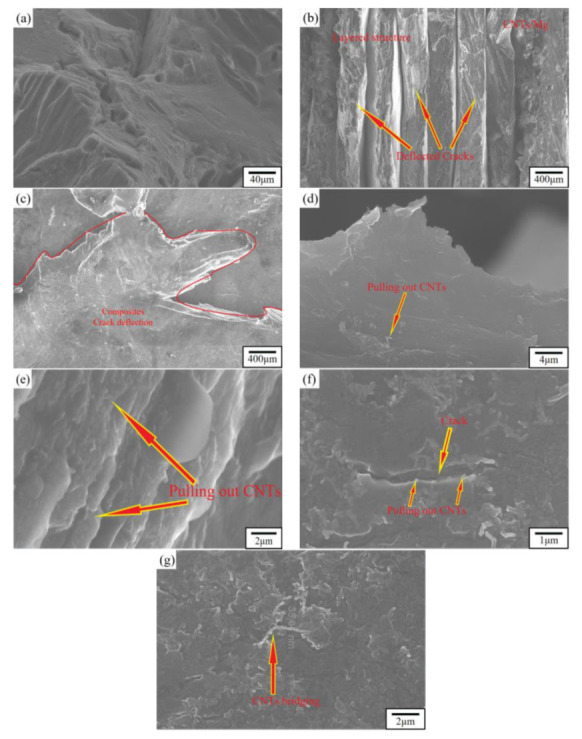
SEM image of Mg and CNTs/Mg composites: (**a**) the fracture surface of a single piece of Mg shows obvious cleavage fracture; (**b**) the fracture surface of the CNTs/Mg composite shows the deflection crack propagation of the CNT layer; (**c**) traces of crack deflection in the CNTs/Mg composite; (**d**,**e**) pull-out CNTs from the fracture; (**f**) pull-out CNTs from cracks; (**g**) carbon nanotube bridging and connecting Mg foil.

The crack surfaces of pure Mg and the CNTs/Mg composites are shown in Figure 9. Because the plasticity of Mg is limited, the crack surface of the pure Mg tensile sample is shown in Figure 9a. The crack morphology of micro nanolayered CNTs/Mg composites prepared by the above method changed greatly compared with pure Mg, from smooth fractures to rough serrated fractures as shown in Figure 9b,c. The CNT layer not only acts as a high-strength barrier against crack propagation [23] but also effectively inhibits grain growth as shown in Figure 6. The layered structure not only delays crack initiation in the composite but also inhibits crack propagation [29,30,31] as shown in Figure 9b,c.

In the CNTs/Mg composites, “CNT pull out”, “crack deflection”, and “CNT bridge” were ubiquitous. The CNTs were drawn from the Mg matrix as shown in Figure 9d–f. On the one hand, because the CNTs still maintained a certain integrity after pretreatment and sintering, which was effective in improving the strength and ductility of the composites, the CNTs had a higher specific strength and specific stiffness, requiring more energy to pull them out of the matrix. On the other hand, due to the biomimetic layer construction, the CNT layer separated the Mg matrix layer and adopted SPS, which could effectively prevent the excessive grain size in the Mg matrix, and the agglomeration of CNTs could also be reduced by deposition of CNTs on Mg foil by electrophoresis. According to the observation of tensile morphology and the above description, the CNTs/Mg layer structure significantly changed the fracture behavior of the composites. The layered structure of CNTs/Mg was shown to release the stress concentration at the crack tip and hinder crack propagation. Deflection and bifurcation of cracks were also observed, and then the path of crack propagation became a zigzag as shown in Figure 9c. All these factors significantly increased the energy absorbed by crack propagation and improved the ductility of composites. At the crack propagation stage, the deflection and bifurcation of cracks caused by layered structures improved the ductility of composites.

### 3.4. Damping Properties of CNTs/Mg Laminated Composites

#### 3.4.1. Effect of Temperature on the Damping Properties of CNTs/Mg Composites

**Figure 10:**
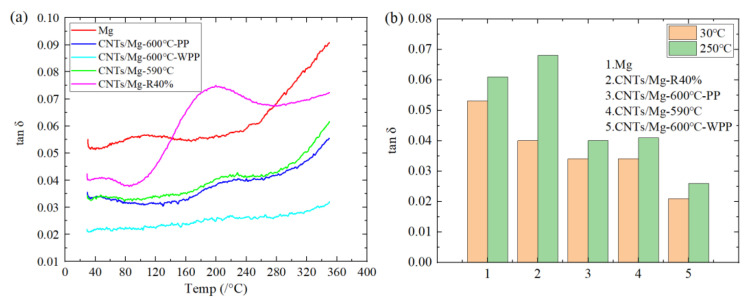
Effects of different preparation processes on damping properties: (**a**) temperature-dependent damping curves (tanδ~T) and (**b**) room temperature (25 °C) and high temperature (250 °C) damping capacity at ƒ = 1 Hz.

Figure 10a shows the damping–test temperature curve (tanδ~T) of pure Mg and CNTs/Mg composites at a vibration frequency of 1 Hz. The damping value of layered Mg was almost independent of the test temperature in the range of room temperature to 230 °C, but it increased slowly in the high-temperature zone (240–350 °C). The damping value of CNTs/Mg sintered at 600 °C was almost independent of the test temperature in the temperature range of room temperature to 170 °C, but it increased slowly in the medium temperature range (i.e., 170–240 °C). The test temperature had little effect on the damping value at 240–280 °C, and it increased slowly at 280–350 °C. By comparing the two curves, the damping performance of the method of holding pressure to room temperature after holding temperature and pressure can be seen to be better. The trend of CNTs/Mg composites sintered at 590 and 600 °C was consistent with the above situation, but the samples sintered at 590 °C had better damping properties. The damping value of the sample sintered at 590 °C and then rolled 40% was better than the damping value of the unrolled composite, even exceeding pure Mg in some test temperature ranges. However, the test tanδ~T curve was quite different from the test tanδ~T curve of the above composite. In the range of room temperature to 100 °C, the damping value produced only an internal friction peak at approximately 50 °C, and the damping value had nothing to do with the test temperature. The damping value rose at 100–200 °C and formed an internal friction peak at approximately 200 °C. Then, the damping value began to decline slowly, and the damping value began to rise slowly until 280 °C. Comparing the data before and after rolling of the sample shows that the damping property after rolling was better. By testing pure Mg and CNTs/Mg composites, after adding CNTs, the damping value of the composite can be seen to decrease, and the damping value gradually increased with increasing test temperature.

Figure 10b shows the damping properties of the CNTs/Mg composites at different sintering temperatures, pressurization methods, and rolling 40% at room temperature (30 °C) and high temperature (250 °C). The damping value of samples sintered at 600 °C after holding to room temperature and removing the pressure after holding to room temperature increased by 62% at room temperature and 54% at high temperature. Compared with the samples sintered at 590 and 600 °C, the damping value changed little at room temperature or high temperature. The damping performance of 40% of the rolled samples was higher than the damping performance of the non-rolled samples, both at room temperature and high temperature. Compared with pure Mg, the damping value of composites with CNTs was lower, but the damping value of the samples after the first rolling at high temperature was higher than the damping value of pure Mg.

By analyzing the CNTs/Mg composites at different test temperatures tan δ-T of the curves, in Figure 10a, the CNTs/Mg composites prepared by the above method not only refined the grains of the Mg matrix compared with other preparation methods but also after EPD of CNTs on Mg foil, the dislocations in the composites were pinned by CNTs, and the dislocations were entangled and stuffed as shown in Figure 8c. The length of dislocations was not only greatly reduced, but it also greatly reduced the number of movable dislocations. Under the action of external stress, internal friction (P_1_) can only be generated by the resonant motion of dislocated strings, so the damping performance of the composite was not affected by temperature [32]. At 220–240 °C, the internal friction peak (P_2_) as caused by the interfacial damping mechanism between the matrix and matrix and between the matrix and the reinforcing phase. When the test temperature increased to 220 °C, the dislocation began to break away from the weak pinpoint but was still pinned by the strong pinpoint. When the dislocation reached its peak temperature, an avalanche of nailing will occur, the original solid bonding strength between the matrix and enhanced phase begins to weaken with increasing temperature, and the relative slip between interfaces of adjacent particles is more likely to occur, resulting in friction and internal friction [33,34]. However, there are few slip systems in Mg with a hexagonal closest packed (HCP) structure at room temperature. When the temperature reached 240 °C, new slip surfaces appeared in Mg. Due to the increase in slip systems, the number of movable dislocations increased greatly, and the damping performance of composites increased rapidly after this transition point [35].

#### 3.4.2. Effect of Frequency on the Damping Properties of CNTs/Mg Composites

**Figure 11:**
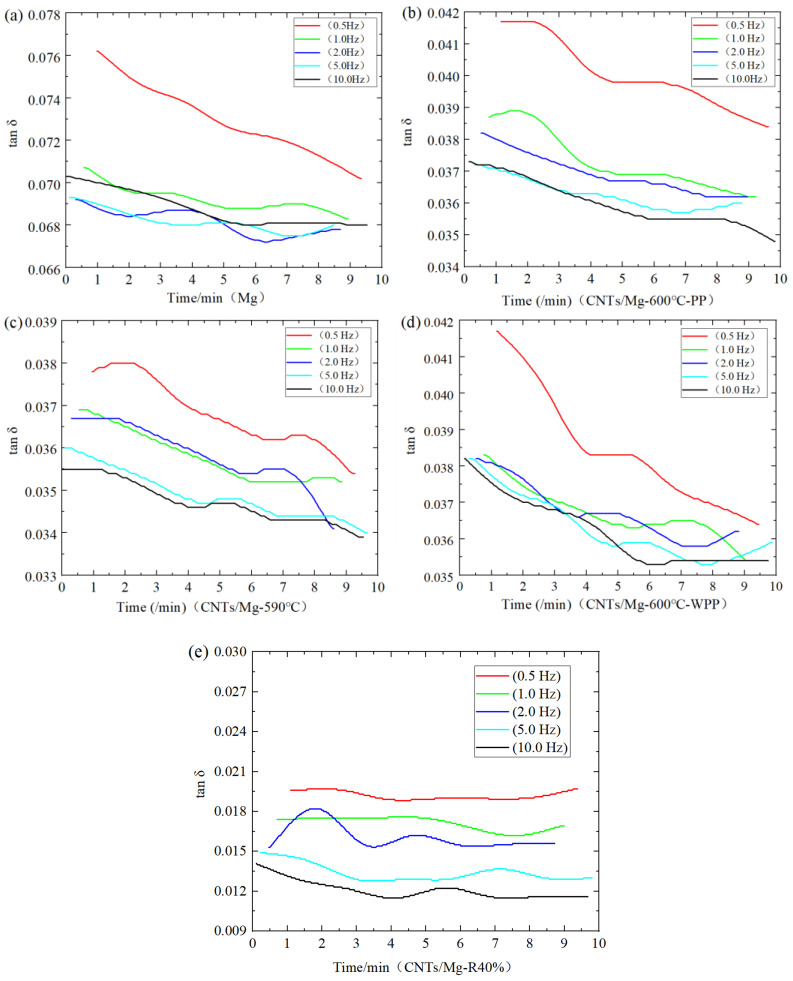
The tan δ~time curve of the base material and the CNTs/Mg composite at different frequencies. (**a**) Pure Mg; (**b**) 600 °C sintering; (**c**) 590 °C sintering; (**d**) 600 °C sintering without preserving pressure after the end of holding time; (**e**) 590 °C sintering after the pass 40% hot rolling.

Figure 11 shows the tan δ~time curves of the pure Mg and CNTs/Mg composites at different frequencies. When the strain amplitude was 30 × 10^−6^ at room temperature, as the frequency increased, the damping performance of the material decreased. The damping performance at 0.5 Hz was higher than the damping performance at 1.0–10 Hz. While the damping of 1.0–10 Hz decreased slightly with increasing vibration frequency, the damping decreased very little compared with the decrease of 0.5–1 Hz. At the same frequency, the damping performance of Figure 11a–d gradually decreased with increasing vibration time, while the damping value of Figure 10e hardly changed with time. There was only a peak at 2.0 Hz, and the curves at other frequencies were almost flat. The damping value of CNTs/Mg sintered at 600 °C was not much different when the temperature was maintained, and the pressure was maintained at room temperature. The sample sintered at 590 °C had the same damping value. The damping value of the sample after 590 °C sintering and 40% rolling was the lowest. The damping value of the composite material with CNTs added above was lower than that of the matrix material.

When the samples were tested at room temperature, the greater the frequency, the smaller the damping value of the CNTs/Mg composites, possibly because the damping of CNTs/Mg composites at room temperature was mainly dislocation damping, which was still in the stage of low strain amplitude. On the one hand, the external vibration caused dislocation movement. Because the impurity atoms were pinned on the dislocation line, the dislocation created string resonance movement to produce internal friction, but the dislocation did not come off the nail. However, when the shear stress at the interface is large enough to overcome the friction resistance, interface slip may occur, and the friction energy loss caused by interface slip is one of the important factors for damping [16]. In addition, at the same stress level, interface slip can be carried out more thoroughly at a low frequency than at a high frequency, because the frequency is the reciprocal of the stress cycle time, which leads to an increase in damping with a decrease in frequency [36].

## 4. Conclusions

(1) At a voltage of 300 V, the EPD time was a decisive factor affecting the deposition morphology. SEM was used to observe the deposition morphology of CNTs on the surface of Mg foil with different deposition times (i.e., 0.5, 1, and 1.5 min) under 300 V. The CNT layer stack was denser, and some CNTs formed overlaps and still retained enough gaps. The time for good deposition was 0.5 min;
(2) Two damping peaks, P_1_ and P_2_, were observed in the temperature-dependent damping spectrum of CNTs/Mg composites. Peak P_1_ is thought to be related to the interaction between dislocations and impurity atoms or vacancies. Peak P_2_ is believed to be caused by grain boundary sliding. The damping properties tested at room temperature decreased gradually with increasing frequency;
(3) Compared with layered Mg, the strength and elongation of biomimetic layered CNTs/Mg composites were improved. After 40% rolling, the layered CNTs/Mg composites were much higher than pure Mg. After heat preservation, the grain size of the method of holding pressure to room temperature was larger than the grain size of the method of not holding pressure to room temperature, but the wettability of the CNT layer and Mg layer was poor. To prevent the dispersion of the layered structure and effectively carry out the subsequent preparation process, the method of holding pressure to room temperature was still selected in the preparation process. The CNTs/Mg layered structure showed a good toughening effect. Micro nanostructures balanced the conflict between the strength and toughness of Mg matrix composites. Compared with Mg matrix composites prepared by other processes, CNTs/Mg layered composites could not only achieve a higher strengthening effect but also had an excellent toughening effect.

## Figures and Tables

**Table 1:** Mechanical properties of Mg and laminated CNTs/Mg composites.

Materials	YS (MPa)	UTS (MPa)	ε_f_ (%)
Mg	44.88	73.59	1.75
CNTs/Mg (590 °C)	40.87	86.10	3.33
CNTs/Mg (600 °C-WPP)	39.14	65.64	2.50
CNTs/Mg (600 °C-PP)	45.40	79.70	2.017
CNTs/Mg (R40%)	143.97	185.57	2.34
